# Efficacy and Safety of Glutathione Supplementation in Patients with HIV Infection and HIV-Tuberculosis Co-Infection

**DOI:** 10.3390/nu18040571

**Published:** 2026-02-09

**Authors:** John Dawi, Scarlet Affa, Stefanie Au, Yura Misakyan, Edgar Gonzalez, Abraham Chorbajian, Mary Hammi, Priyanka Dave, Kyla Qumsieh, Vishwanath Venketaraman

**Affiliations:** 1 College of Osteopathic Medicine of the Pacific, Western University of Health Sciences, Pomona, CA 91766, USA; john.dawi@westernu.edu (J.D.); stefanie.au@westernu.edu (S.A.); yura.misakyan@westernu.edu (Y.M.); edgar.gonzalez@westernu.edu (E.G.); abraham.chorbajian@westernu.edu (A.C.); mary.hammi@westernu.edu (M.H.); priyanka.dave@westernu.edu (P.D.); kyla.qumsieh@westernu.edu (K.Q.); 2 Department of Biochemistry and Chemistry, The College of UCLA, 405 Hilgard Avenue, Los Angeles, CA 90095, USA; scarleta@ucla.edu

**Keywords:** glutathione, oxidative stress, HIV infection, NAC, tuberculosis (TB)

## Abstract

Glutathione (GSH), the most abundant intracellular non-protein thiol, is a central regulator F redox homeostasis, immune function, and mitochondrial integrity. In human immunodeficiency virus (HIV) infection, persistent oxidative stress and impaired precursor availability result in sustained glutathione deficiency, contributing to immune dysfunction, inflammation, and disease progression despite effective antiretroviral therapy. This redox imbalance is further exacerbated in HIV–tuberculosis co-infection, where compounded inflammatory and metabolic stress increases susceptibility to opportunistic infections and treatment-related complications. This review examines the efficacy and safety of glutathione supplementation and precursor-based strategies in HIV infection and HIV–tuberculosis co-infection. Evidence from mechanistic studies, clinical trials, and translational research suggests that glutathione repletion, achieved through direct supplementation or precursor approaches such as *N*-acetylcysteine, Glycine and *N*-acetylcysteine (GlyNAC), and cysteine-rich dietary interventions, can restore intracellular thiol balance, improve immune cell function, enhance mitochondrial performance, and reduce systemic oxidative stress. These interventions have shown consistent safety and tolerability across diverse populations, including individuals receiving complex antiretroviral and antitubercular regimens, with gastrointestinal discomfort being the most commonly reported adverse effect and serious toxicities remaining rare. Despite encouraging findings, translation into routine clinical practice remains limited by methodological heterogeneity, short study durations, and lack of standardized biomarkers and long-term outcome data. Future research should prioritize rigorously designed trials incorporating mechanistic endpoints, standardized redox measurements, and clinically meaningful outcomes. Collectively, the available evidence supports glutathione-centered strategies as promising adjuncts to existing HIV and tuberculosis treatment paradigms, warranting further investigation to define their role in improving immune resilience and long-term clinical outcomes.

## 1. Introduction

Human immunodeficiency virus (HIV) infection is characterized by persistent immune activation, chronic inflammation, and sustained oxidative stress, all of which contribute to progressive immune dysfunction and multisystem complications. Central to these processes is disruption of cellular redox homeostasis, driven in large part by depletion of intracellular glutathione. Glutathione is the most abundant intracellular non-protein thiol and functions as a primary antioxidant, maintaining redox balance by neutralizing reactive oxygen species, preserving protein thiol integrity, and supporting enzymatic detoxification pathways essential for cellular survival. Perturbations in glutathione metabolism alter redox-sensitive signaling cascades, impair immune cell function, and promote oxidative stress-mediated tissue injury [1].

Beyond its antioxidant role, glutathione is a critical regulator of immune competence. Adequate intracellular glutathione levels are required for normal lymphocyte activation, cytokine production, and resistance to apoptosis. Experimental and clinical studies have demonstrated that glutathione depletion compromises both innate and adaptive immune responses, linking redox imbalance directly to immune dysregulation [2]. In chronic disease states, including HIV infection, this disruption of redox control contributes to sustained inflammation and progressive immune exhaustion.

Multiple investigations have established that glutathione deficiency is a consistent biochemical abnormality in individuals living with HIV. Reduced intracellular glutathione concentrations have been documented in peripheral blood mononuclear cells, CD4+ T lymphocytes, and macrophages, reflecting both increased oxidative consumption and impaired antioxidant reserve within key immune compartments [3,4]. Importantly, this deficiency is not transient. Rather, it persists across disease stages and is reinforced by ongoing immune activation and inflammatory signaling.

The clinical relevance of glutathione depletion in HIV extends beyond laboratory findings. Seminal work by Herzenberg and colleagues shows that lower intracellular glutathione levels are associated with impaired survival in HIV disease, establishing glutathione status as a prognostically significant determinant rather than a passive epiphenomenon [5]. This observation provided early clinical evidence that oxidative stress and redox imbalance actively contribute to disease progression and adverse outcomes in HIV-infected populations.

Mechanistically, impaired glutathione homeostasis in HIV is driven in part by limitations in biosynthetic capacity. Glutathione synthesis depends on the availability of cysteine, the rate-limiting amino acid in the glutathione synthetic pathway. HIV infection is associated with altered amino acid metabolism and reduced sulfur amino acid availability, constraining endogenous glutathione production and preventing effective replenishment of intracellular stores [6]. In the context of chronic oxidative stress, this substrate limitation further amplifies glutathione depletion and perpetuates redox imbalance.

Glutathione deficiency has particularly important implications for host defense against opportunistic infections. Effective immune control of intracellular pathogens requires intact macrophage antimicrobial activity and robust Th1-polarized cytokine responses. Experimental studies have shown that glutathione depletion impairs macrophage phagocytosis, intracellular killing, and antigen presentation, weakening immune surveillance and pathogen clearance [4]. In HIV infection, these defects contribute to increased susceptibility to opportunistic infections, including tuberculosis, and exacerbate inflammatory tissue injury.

Notably, glutathione deficiency and oxidative stress often persist despite effective antiretroviral therapy. While viral suppression reduces some drivers of immune activation, residual inflammation and mitochondrial dysfunction continue to sustain redox imbalance [2]. This persistence highlights a gap in current treatment paradigms and underscores the need for adjunctive strategies that specifically target glutathione depletion and oxidative stress rather than relying on antiretroviral therapy alone to restore redox homeostasis.

Taken together, these observations establish glutathione depletion as a central and clinically meaningful feature of HIV pathophysiology. By linking redox imbalance to immune dysfunction, susceptibility to opportunistic infection, and survival outcomes, the existing evidence provides a strong rationale for therapeutic strategies aimed at restoring intracellular glutathione levels. These strategies, including direct supplementation and precursor-based interventions, form the focus of the sections that follow. Although supportive, the evidence is not yet definitive, emphasizing the need for large well-controlled clinical trials to further investigate this idea.

## 2. Background

The persistent glutathione deficiency observed in HIV infection reflects a convergence of biochemical, metabolic, and immunologic disturbances that extend beyond acute viral replication. As established in Section 1, oxidative stress is sustained even in individuals receiving effective antiretroviral therapy, indicating that redox imbalance is maintained by host–pathogen interactions and chronic immune activation rather than viremia alone. This background section delineates the mechanistic pathways through which HIV disrupts glutathione homeostasis and clarifies why redox failure remains a central feature of disease progression.

One of the earliest and most consistent findings in HIV-associated oxidative stress is dysregulation of thiol redox balance. HIV infection shifts intracellular redox states toward oxidized glutathione, reducing the availability of reduced glutathione required for antioxidant defense and redox signaling. Staal and others demonstrated that HIV is associated with both absolute depletion of reduced glutathione and accumulation of oxidized glutathione, reflecting impaired redox cycling rather than simple deficiency alone [7]. This altered redox ratio compromises cellular signaling pathways that depend on reversible thiol modifications, particularly within immune cells.

At the cellular level, glutathione depletion disrupts immune cell survival and function through redox-sensitive control mechanisms. Lymphocytes require adequate glutathione to maintain mitochondrial membrane integrity and regulate apoptosis. Experimental studies have shown that HIV-infected individuals exhibit increased lymphocyte apoptosis associated with thiol depletion, linking redox imbalance to progressive immune cell loss independent of direct viral cytotoxicity [8]. These findings provided early mechanistic insight into how oxidative stress accelerates CD4+ T-cell depletion beyond viral replication alone.

Macrophages are similarly affected by glutathione insufficiency. As central effectors of innate immunity, macrophages rely on glutathione to support phagocytosis, intracellular pathogen killing, and antigen presentation. In HIV infection, macrophage glutathione depletion impairs these antimicrobial functions, reducing intracellular control of pathogens and amplifying inflammatory signaling [9]. Restoration of glutathione in experimental systems has been shown to reverse many of these defects, reinforcing the causal role of redox imbalance in macrophage dysfunction.

Cytokine dysregulation represents another critical consequence of impaired glutathione metabolism. HIV-associated oxidative stress skews cytokine production away from Th1-polarized responses toward a more inflammatory yet less effective immune profile. Glutathione depletion suppresses interleukin-2 and interferon-gamma production while permitting excessive tumor necrosis factor-alpha and interleukin-8 signaling, contributing to immune exhaustion and chronic inflammation [8]. These alterations weaken adaptive immune responses and impair coordination between innate and adaptive immunity.

The role of sulfur amino acid metabolism in sustaining glutathione deficiency has become increasingly apparent. HIV infection alters amino acid availability through increased metabolic demand, inflammation-driven catabolism, and impaired absorption. Clinical studies observed reduced plasma concentrations of cysteine and related sulfur-containing amino acids in HIV-infected individuals, establishing substrate limitation as a key contributor to impaired glutathione synthesis [10]. Without adequate precursor availability, endogenous glutathione production cannot keep pace with oxidative consumption.

Intervention studies targeting sulfur amino acid replenishment further support this mechanism. Supplementation with alpha-lipoic acid, a compound that enhances cysteine availability and redox cycling, has been shown to restore total blood glutathione levels and improve lymphocyte function in patients with HIV [11]. These findings provided direct clinical evidence that correcting precursor deficiency can partially reverse immune dysfunction linked to glutathione depletion.

Dietary supplementation studies reinforce the importance of substrate availability. Borges-Santos and colleagues demonstrated that supplementation with cysteine or glutamine significantly increased plasma glutathione concentrations in HIV-positive individuals, with differential responses depending on baseline metabolic status [12]. These results confirmed that impaired glutathione synthesis in HIV is not irreversible and can be modulated through targeted nutritional interventions.

Beyond single-nutrient approaches, broader sulfur supplementation strategies have also been evaluated. Randomized trials of sulfur-containing compounds showed improvements in immune function, including enhanced lymphocyte proliferation and cytokine responsiveness, supporting the concept that restoring thiol availability improves redox-sensitive immune processes [13,14]. These studies expanded the therapeutic framework beyond pharmacologic antioxidants toward metabolic repletion strategies.

**Figure 1:**
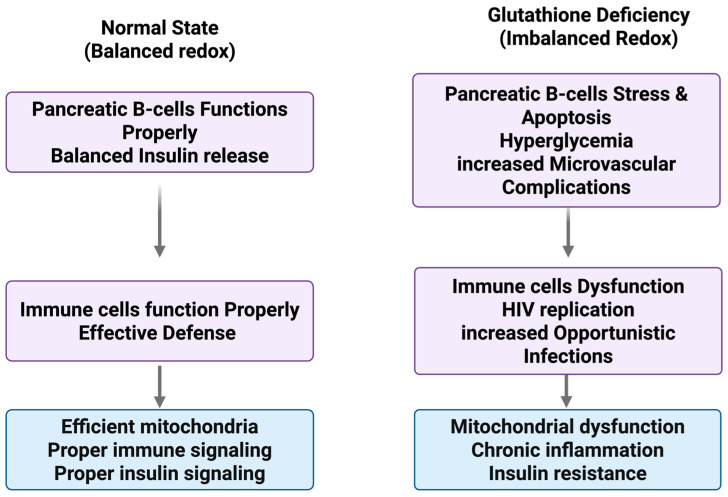
Pathophysiological Roles of Glutathione Deficiency in HIV and T2DM.

Collectively, these background findings establish HIV-associated glutathione deficiency as a multifactorial process driven by interconnected redox, inflammatory, and metabolic disturbances [Figure 1]. Importantly, they demonstrate that glutathione depletion is biologically actionable. The ability to restore glutathione levels through precursor and sulfur-based interventions provides the mechanistic foundation for therapeutic strategies examined in subsequent sections.

## 3. Glutathione Supplementation and Precursor Strategies in HIV and HIV–Tuberculosis Co-Infections

Given the established role of glutathione depletion in HIV-associated immune dysfunction and oxidative stress, direct glutathione supplementation and precursor-based strategies have been extensively investigated as adjunctive therapeutic approaches. These interventions aim to restore intracellular thiol balance, improve immune cell function, and mitigate oxidative injury that persists despite effective antiretroviral therapy. Among these strategies, pharmacologic glutathione precursors have received the greatest attention due to their bioavailability, mechanistic rationale, and favorable safety profiles.

One of the most influential studies examining glutathione precursor therapy in HIV was conducted by De Rosa and colleagues, who demonstrated that *N*-acetylcysteine supplementation effectively replenished intracellular glutathione levels in lymphocytes from HIV-infected individuals [15]. This study provided direct evidence that cysteine availability is a limiting factor in glutathione synthesis in HIV. By supplying cysteine in a stable, bioavailable form, NAC restored intracellular glutathione pools, with corresponding improvements in immune-related biomarkers, including enhanced lymphocyte viability and redox-sensitive signaling, establishing that precursor therapy exerts biologically meaningful effects beyond biochemical normalization [15].

Building upon these mechanistic findings, Gupta et al. evaluated NAC supplementation in a randomized, placebo-controlled trial involving older HIV-infected adults receiving antiretroviral therapy [16]. Aging represents a state of increased oxidative stress, endothelial dysfunction, and mitochondrial decline, which is compounded by chronic HIV infection. NAC administration resulted in measurable improvements in endothelial function, as assessed by flow-mediated dilation, alongside reductions in systemic oxidative stress markers. These findings were significant because cardiovascular disease has emerged as a leading cause of morbidity in virally suppressed HIV populations. The study demonstrated that glutathione precursor therapy can influence vascular biology, linking redox modulation to clinically relevant outcomes [16].

Combination strategies targeting multiple components of glutathione metabolism have also been explored. Look and colleagues conducted a randomized trial combining NAC with sodium selenite, a critical cofactor for glutathione peroxidases [17]. This dual approach addressed both glutathione synthesis and enzymatic utilization. The intervention resulted in improved antioxidant capacity and enhanced lymphocyte proliferation compared with placebo. These findings emphasized that glutathione metabolism operates within an interconnected network of micronutrients and enzymes, and that isolated precursor supplementation may be less effective than coordinated metabolic support [17].

More recently, attention has shifted toward multi-precursor formulations designed to overcome multiple rate-limiting steps in glutathione synthesis. Kumar et al. evaluated supplementation with glycine plus NAC (GlyNAC) in HIV-infected individuals and demonstrated improvements across a broad range of biological domains [18]. GlyNAC supplementation increased intracellular glutathione levels and improved multiple metabolic and functional parameters. Functional outcomes included improvements in insulin sensitivity, muscle strength, and cognitive performance. These findings were particularly notable because they linked glutathione repletion to reversal of aging-related phenotypes that are disproportionately prevalent in treated HIV populations [18].

The relevance of glutathione precursor strategies extends beyond HIV monoinfection to HIV–tuberculosis co-infection, where oxidative stress is further amplified. Safe and colleagues conducted an open-label, randomized phase II trial evaluating NAC as an adjunctive therapy in hospitalized patients with HIV-associated tuberculosis [19]. NAC supplementation was safe and well tolerated in conjunction with antiretroviral and antitubercular therapy. Although the study was not powered for definitive clinical endpoints, trends toward reduced oxidative stress and improved inflammatory profiles were observed, supporting the biological plausibility of glutathione repletion in this high-risk population [19].

Follow-up analyses from the same investigative group further show that adjunctive NAC therapy dampened oxidative stress markers in peripheral blood of patients with HIV-associated tuberculosis [2]. These findings reinforced the concept that redox imbalance is a modifiable contributor to disease severity in co-infected individuals. Importantly, NAC did not interfere with antimicrobial efficacy or drug metabolism, addressing a critical concern in patients receiving complex multidrug regimens [2].

Collectively, these studies establish that glutathione supplementation and precursor-based strategies can restore intracellular thiol balance, improve immune and endothelial function, and attenuate oxidative stress in HIV infection. The extension of these findings to HIV–tuberculosis co-infection highlights the translational relevance of redox-targeted interventions in settings of compounded inflammatory and metabolic stress. These data provide a strong mechanistic and clinical foundation for continued investigation of glutathione-centered therapies as adjuncts to standard HIV and TB treatment paradigms.

## 4. Cysteine-Based Diet Changes in HIV Infection

In addition to pharmacologic glutathione precursors, dietary strategies aimed at increasing cysteine availability have been investigated as a means of restoring glutathione homeostasis in HIV-infected populations. Nutritional insufficiency is common in HIV infection due to altered metabolism, chronic inflammation, gastrointestinal dysfunction, and increased oxidative demand. Because cysteine is the rate-limiting substrate for glutathione synthesis, dietary interventions that increase cysteine intake represent a biologically plausible and clinically accessible approach to addressing redox imbalance.

One of the earliest lines of evidence supporting dietary cysteine augmentation comes from studies evaluating whole-protein supplementation. Micke and colleagues demonstrated that oral supplementation with cysteine-rich whey protein significantly increased plasma glutathione concentrations in HIV-infected individuals [11]. Whey protein is naturally enriched in cysteine-containing amino acids and provides substrates necessary for endogenous glutathione synthesis. The study showed that dietary protein alone, without pharmacologic intervention, could measurably enhance systemic glutathione availability. Importantly, supplementation was well tolerated and did not interfere with antiretroviral therapy, highlighting its feasibility as a supportive nutritional strategy [11].

Subsequent investigations extended these findings to longer intervention periods. In a follow-up study, long-term whey protein supplementation maintained elevated plasma glutathione levels in patients with HIV over time [20]. This finding was clinically relevant because HIV-associated oxidative stress is chronic rather than transient. Sustained improvement in glutathione status suggests that dietary cysteine-based interventions can provide ongoing redox support rather than short-lived biochemical correction. The study also demonstrated that nutritional interventions can be integrated into long-term HIV care without loss of efficacy or tolerability [20].

Beyond protein-based supplementation, targeted amino acid interventions have provided additional insight into cysteine metabolism in HIV. Clinical trials assessing sulfur amino acid supplementation have shown that increasing cysteine availability improves immune function, including lymphocyte proliferation and cytokine responsiveness [1]. These effects are consistent with the known dependence of immune cell activation and survival on intracellular glutathione levels. By restoring cysteine supply, these dietary strategies support glutathione synthesis and redox-sensitive immune signaling pathways that are disrupted in HIV infection [1].

Metabolic studies further underscore the relevance of cysteine deficiency in HIV. Ziegler and colleagues demonstrated that HIV-infected youth exhibit significantly reduced concentrations of sulfur-containing amino acids, including cysteine, compared with healthy controls [21]. These deficiencies were associated with markers of immune dysfunction and inflammation, establishing cysteine depletion as a metabolic hallmark of HIV infection rather than a secondary nutritional artifact. This work provided strong evidence that dietary insufficiency contributes directly to impaired glutathione synthesis and redox imbalance in HIV [21].

The implications of cysteine-based dietary strategies extend to HIV–tuberculosis co-infection. Tuberculosis imposes additional oxidative and metabolic stress, further increasing glutathione consumption and cysteine demand. In co-infected individuals, dietary cysteine insufficiency may be exacerbated, compounding immune dysfunction and impairing host defense against Mycobacterium tuberculosis. Although direct dietary intervention trials in HIV–TB populations remain limited, the shared redox mechanisms support extrapolation of these findings to co-infected settings [11,12,13,14,15,16,17,19,20,21].

Collectively, these studies demonstrate that cysteine-based dietary interventions can meaningfully enhance glutathione availability, support immune function, and provide sustained redox stabilization in HIV infection. Unlike pharmacologic supplementation, dietary strategies offer a low-cost, scalable approach that can be integrated into comprehensive care models, particularly in resource-limited settings. These findings position cysteine-rich nutrition as a complementary component of glutathione-centered interventions in HIV and potentially in HIV–tuberculosis co-infection.

## 5. NAC Therapy in HIV-Tuberculosis Co-Infection

HIV–tuberculosis co-infection represents a clinical setting characterized by extreme oxidative stress, heightened inflammatory burden, and compounded immune dysfunction. Both HIV and Mycobacterium tuberculosis independently disrupt redox homeostasis through sustained production of reactive oxygen species, mitochondrial injury, and inflammatory cytokine signaling. When these infections coexist, oxidative stress is amplified, accelerating glutathione depletion and further impairing immune containment of intracellular pathogens. In this context, NAC has emerged as a rational adjunctive therapy aimed at restoring thiol balance and supporting immune and organ function.

Clinical evaluation of NAC in HIV–tuberculosis co-infection was advanced by Safe and colleagues, who conducted an open-label randomized phase II trial assessing oral NAC in hospitalized patients with HIV-associated tuberculosis [22]. The study demonstrated that NAC supplementation was safe and feasible when administered alongside standard antitubercular and antiretroviral regimens. Importantly, no clinically significant drug–drug interactions or treatment-limiting toxicities were observed. Biomarker analyses revealed trends toward reduced oxidative stress and improved redox balance, supporting the hypothesis that glutathione precursor therapy can partially counteract the oxidative burden imposed by co-infection [22].

Follow-up analyses from the RIPENACTB study further clarified NAC’s biological impact in this population. Safe et al. reported that adjunctive NAC therapy significantly dampened oxidative stress markers in peripheral blood, indicating effective systemic thiol replenishment [23]. These findings were notable because oxidative stress in HIV–TB co-infection is associated with immune dysregulation, tissue damage, and poor treatment outcomes. The ability of NAC to reduce oxidative biomarkers without compromising antimicrobial efficacy strengthened its candidacy as a supportive therapy in co-infected patients [23].

Beyond redox modulation, NAC’s influence on immune cell survival has important implications for HIV–TB management. Treitinger and colleagues demonstrated that NAC supplementation reduced lymphocyte apoptosis and improved intracellular thiol status in HIV-infected individuals receiving antiretroviral therapy [24]. Excessive immune cell apoptosis contributes to impaired immune reconstitution and vulnerability to opportunistic infections. By preserving lymphocyte viability, NAC may enhance immune resilience in co-infected individuals facing dual pathogen stress [24].

Hepatotoxicity is a major clinical challenge in HIV–tuberculosis co-infection due to the combined hepatic burden of antiretroviral and antitubercular drugs. Moosa et al. evaluated intravenous NAC for the management of anti-tuberculosis drug-induced liver injury in a randomized controlled trial that included a high proportion of HIV-infected participants [25]. NAC administration was associated with improved liver function recovery and was well tolerated, reinforcing glutathione’s central role in hepatic detoxification pathways. This study demonstrated that NAC not only addresses immune and redox dysfunction but also mitigates treatment-related organ toxicity in co-infected populations [25].

Mechanistic context for these clinical findings is provided by broader immunologic studies of glutathione metabolism. Dröge and Breitkreutz emphasized that glutathione availability is a determinant of T-cell activation, cytokine balance, and immune competence [26]. NAC supplementation restores intracellular thiol pools necessary for redox-sensitive signaling pathways, thereby correcting immune defects exacerbated by HIV–TB co-infection. This mechanistic framework supports the observed clinical and biomarker improvements seen in NAC trials [26].

Additional randomized studies examining sulfur-based interventions further corroborate NAC’s immunomodulatory role. Breitkreutz and colleagues demonstrated that sulfur supplementation improved immune function and lymphocyte responsiveness in HIV-infected individuals [27]. Although not limited to tuberculosis, these findings reinforce the principle that restoring thiol availability enhances immune capacity in settings of chronic oxidative stress. In HIV–TB co-infection, where immune exhaustion is pronounced, such redox-targeted interventions may be particularly beneficial [27].

Collectively, these studies establish NAC as a biologically plausible and clinically feasible adjunctive therapy in HIV–tuberculosis co-infection. NAC supplementation restores glutathione availability, reduces oxidative stress, supports immune cell survival, and protects against drug-induced organ injury without compromising standard antimicrobial therapy. These findings justify continued investigation of NAC within integrated treatment frameworks for co-infected populations.

## 6. Safety and Tolerability of Glutathione Supplementation and Precursors

As glutathione-centered interventions are considered for chronic use in HIV infection and HIV–tuberculosis co-infection, safety and tolerability represent critical determinants of clinical feasibility. Individuals living with HIV are frequently exposed to lifelong antiretroviral therapy, intermittent antimicrobial regimens, and medications for comorbid conditions, raising legitimate concerns regarding drug interactions, cumulative toxicity, and long-term adverse effects. Accordingly, multiple studies and reviews have systematically evaluated the safety profile of glutathione supplementation and its precursors, particularly NAC [Table 1].

Early clinical experience established that NAC is well tolerated in HIV-infected populations. Atkuri and colleagues reviewed NAC use across diverse clinical settings and emphasized its favorable safety profile as a cysteine donor and glutathione precursor [28]. Importantly, NAC does not function as a nonspecific antioxidant scavenger but rather restores endogenous glutathione synthesis, preserving physiologic redox signaling. Across studies, adverse effects were predominantly mild and gastrointestinal in nature, including nausea, bloating, and diarrhea, with low discontinuation rates even during prolonged administration [28].

Broader evaluations of NAC’s clinical utility further support its safety. Tenório et al. conducted an extensive review of NAC use across human health conditions and reported consistent tolerability across dosing regimens and disease states [21]. Oral NAC was associated primarily with transient gastrointestinal symptoms, while serious adverse reactions were rare. Importantly, no evidence of cumulative toxicity, hepatotoxicity, nephrotoxicity, or hematologic abnormalities was observed, even with long-term use. These findings are particularly relevant for HIV populations in whom organ reserve may be compromised [21].

Large-scale reviews examining NAC in oxidative stress-driven disorders provide additional reassurance. Raghu and colleagues analyzed NAC use across pulmonary, cardiovascular, and inflammatory diseases and concluded that NAC exhibits a wide therapeutic window with minimal safety concerns [29]. The review emphasized that NAC’s long history of clinical use, including high-dose and intravenous administration in acute settings, has not revealed significant long-term toxicities. This breadth of experience supports its application in chronic conditions such as HIV, where sustained redox modulation may be required [29].

Schwalfenberg further reinforced NAC’s safety profile in a comprehensive review of its clinical applications [27]. The analysis highlighted NAC’s compatibility with polypharmacy, noting an absence of clinically significant interactions with commonly prescribed medications. This finding is critical in HIV and HIV–tuberculosis co-infection, where treatment regimens are complex and drug–drug interactions pose major challenges. Schwalfenberg also emphasized NAC’s role in hepatic detoxification, further supporting its use in patients exposed to hepatotoxic antiretroviral or antitubercular agents [27].

Safety data are not limited to NAC alone. Direct glutathione supplementation has also been evaluated in human studies. Allen and Bradley assessed oral glutathione supplementation in healthy volunteers and reported no significant adverse effects, with stable hepatic and renal parameters throughout the study period [28]. Although earlier concerns existed regarding oral glutathione bioavailability, subsequent studies demonstrated that supplementation increased systemic glutathione levels without suppressing endogenous synthesis or inducing redox imbalance. These findings broaden the range of safe glutathione repletion strategies beyond precursor-based approaches [28].

Meta-analytic evidence further supports the safety of NAC and related interventions. Faghfouri et al. conducted a systematic review and meta-analysis of controlled clinical trials evaluating NAC’s effects on oxidative stress and inflammatory biomarkers [21]. Across included studies, NAC supplementation did not increase adverse event rates compared with placebo. Serious adverse events were rare, and withdrawal due to intolerance was uncommon. The meta-analysis concluded that NAC is safe across a wide range of doses and clinical contexts, reinforcing its suitability for chronic therapeutic use [21].

In the context of HIV–tuberculosis co-infection, safety considerations are particularly stringent due to overlapping drug toxicities and inflammatory stress. The existing safety data demonstrate that NAC can be safely co-administered with antiretroviral and antitubercular therapies without compromising efficacy or tolerability. The absence of significant hepatic, renal, or hematologic toxicity across studies provides strong reassurance for its integration into complex treatment regimens.

Collectively, these data establish that glutathione supplementation and precursor strategies exhibit a consistently favorable safety and tolerability profile across populations relevant to HIV and HIV–tuberculosis co-infection. Mild gastrointestinal symptoms remain the most commonly reported adverse effects, while serious toxicities are rare. This robust safety foundation supports continued clinical investigation and provides justification for larger, long-term trials evaluating efficacy and implementation in real-world HIV care.

**Table 1:** Glutathione, Glycine, and *N*-acetylcysteine dosage and duration.

Study (Year)	Formulation/Route	Daily Dose	Duration
Richie et al., 2015 [30]	Reduced glutathione (oral)	500 mg or 1000 mg per day	6 months (24 weeks)
Kumar et al., 2023 [18]	GlyNAC (oral)	100 mg/kg each component per day	16 weeks

## 7. Future Directions and Research Gaps

Despite growing interest in glutathione-centered interventions, multiple gaps remain that limit translation into routine clinical care for individuals living with HIV and HIV–tuberculosis co-infection. Current evidence supports biological plausibility and short-term efficacy, yet methodological heterogeneity across studies complicates interpretation and comparison of outcomes. Differences in study duration, formulation, dosing regimens, and outcome measures continue to hinder the development of standardized clinical recommendations [31].

One major limitation is the lack of standardized biomarkers for assessing glutathione status and redox balance. Studies vary widely in their reliance on plasma glutathione, intracellular reduced-to-oxidized glutathione ratios, surrogate oxidative stress markers, or indirect inflammatory endpoints. Without harmonized analytical frameworks, it remains difficult to define clinically meaningful thresholds for glutathione deficiency or repletion. Future trials should incorporate standardized, validated redox biomarkers and longitudinal assessments to determine whether biochemical improvements translate into sustained clinical benefit [31].

Bioavailability and delivery remain additional challenges. Glutathione and its precursors differ substantially in absorption, tissue distribution, and intracellular utilization depending on formulation and route of administration. Emerging delivery strategies, including liposomal glutathione and sustained-release precursor formulations, require direct comparative evaluation to determine relative efficacy and practicality. Interindividual variability driven by diet, genetic polymorphisms, and gut microbiome composition further complicates therapeutic response and underscores the need for stratified or adaptive trial designs [31].

Although *N*-acetylcysteine has been widely used, its pharmacokinetics in chronic disease states remain incompletely characterized. Oral bioavailability is modest, raising questions regarding optimal dosing schedules, formulation, and timing relative to meals and circadian rhythms. Moreover, NAC influences intracellular signaling pathways in addition to serving as a cysteine donor, suggesting that its biological effects may be context dependent. Future studies should integrate transcriptomic and proteomic analyses to better characterize these effects and identify potential off-target signaling consequences [32].

The pleiotropic actions of NAC introduce additional complexity. While antioxidant and anti-inflammatory effects are beneficial in oxidative stress-driven disease, excessive suppression of reactive oxygen species may interfere with physiological signaling pathways essential for immune activation and cellular adaptation. Dose–response relationships are therefore unlikely to be linear. Carefully designed graded-dose studies incorporating mechanistic endpoints are required to define therapeutic windows that restore redox balance without impairing immune competence [33].

Special populations remain underrepresented in existing trials. Pediatric, geriatric, and frail populations may exhibit altered redox biology and distinct responses to glutathione modulation. Given the aging demographics of the HIV population and the increased burden of comorbidities, inclusion of these groups is essential to ensure generalizability and to define age-specific therapeutic strategies [33].

Neurocognitive impairment persists as a significant unmet need in HIV infection despite effective antiretroviral therapy. Central nervous system oxidative stress and mitochondrial dysfunction are increasingly implicated in HIV-associated neurocognitive disorders. Future studies should employ advanced neuroimaging modalities, including magnetic resonance spectroscopy, to quantify brain glutathione levels and correlate them with cognitive performance and neuroinflammatory markers. Pharmacokinetic studies evaluating blood–brain barrier penetration of NAC, GlyNAC, and direct glutathione supplementation are also urgently needed [23].

These considerations are particularly relevant in HIV–tuberculosis co-infection, where systemic and central nervous system oxidative stress may be amplified. Interactions between antiretroviral agents, antitubercular drugs, and redox-targeted therapies require systematic evaluation, particularly given the mitochondrial toxicity associated with certain drug classes. Longitudinal studies incorporating neuroimaging, immune phenotyping, and metabolic endpoints will be essential to define long-term outcomes in co-infected populations [23].

Implementation science represents another critical gap. Adherence, palatability, odor, gastrointestinal tolerability, and cost remain practical barriers to sustained use of glutathione precursors. Comparative trials evaluating sustained-release NAC, GlyNAC, and cysteine-rich dietary proteins are needed to define real-world feasibility. Cost-effectiveness analyses are particularly important in resource-limited settings with high HIV and tuberculosis burden, where scalable adjunctive therapies are most urgently needed [24].

Lessons from psychiatric and neurological trials of NAC further inform future study design. Many prior studies were underpowered and heterogeneous, highlighting the importance of enrichment strategies that select participants with documented glutathione deficiency or elevated oxidative stress markers. Objective mechanistic endpoints should anchor clinical outcomes to reduce placebo effects and improve interpretability. Adaptive trial designs and harmonized adverse-event taxonomies may further enhance efficiency and safety evaluation [34].

Finally, redox biology itself must be approached with nuance. Reactive oxygen species serve essential signaling roles in immune activation and cellular homeostasis. Therapeutic strategies should therefore aim to restore intracellular thiol balance rather than indiscriminately suppress oxidative processes. Within this framework, glutathione-centered interventions align with targeted redox modulation rather than broad-spectrum antioxidant therapy, offering a more physiologically coherent approach to chronic oxidative stress [35].

Recent experience with NAC use in acute inflammatory conditions reinforces its favorable safety profile while emphasizing the importance of timing and disease stage. Early intervention may be more effective than late-stage rescue strategies, a principle that should guide future trials in HIV and HIV–tuberculosis co-infection. Long-term pharmacovigilance and standardized product quality control will be essential as these interventions move toward broader clinical evaluation [29,32].

## 8. Conclusions

Available evidence suggests that glutathione deficiency represents a frequent biochemical and functional abnormality in HIV infection and HIV tuberculosis co-infection, likely driven by interacting oxidative, metabolic, and inflammatory processes. This redox imbalance appears to be associated with dysregulation of both innate and adaptive immune responses, compromised antimicrobial defenses, and markers of disease progression, even in individuals receiving effective antiretroviral therapy. The persistence of oxidative stress despite virologic suppression highlights a potential therapeutic gap that may not be fully addressed by current treatment strategies alone.

Findings from mechanistic, translational, and limited clinical studies indicate that glutathione repletion represents a biologically plausible adjunctive approach. Both direct glutathione supplementation and precursor-based interventions, including *N*-acetylcysteine, GlyNAC, and cysteine-rich dietary strategies, have been associated with improvements in intracellular thiol balance, immune cell function, mitochondrial parameters, and systemic oxidative stress markers. These effects may be particularly relevant in HIV tuberculosis co-infection, where compounded inflammatory and oxidative burdens further impair immune resilience. However, existing studies remain limited in scale, duration, and methodological consistency, underscoring the need for larger, well-controlled trials to clarify optimal dosing, treatment duration, and clinical applicability.

Across available studies, glutathione-centered interventions generally demonstrate favorable safety and tolerability profiles, including in individuals receiving complex antiretroviral and antitubercular regimens, with serious adverse events reported infrequently. While these findings support feasibility for adjunctive use, translation into routine clinical practice remains constrained by heterogeneous study designs, short follow-up periods, and limited long-term outcome data.

Future research should prioritize rigorously designed, adequately powered trials incorporating standardized biomarkers, mechanistic endpoints, and clinically meaningful outcomes. With sufficient methodological rigor and translational focus, glutathione-centered strategies may emerge as valuable adjuncts to existing treatment paradigms, with potential to mitigate persistent oxidative stress, support immune function, and improve long-term outcomes in individuals living with HIV and HIV tuberculosis co-infection.

## 9. Methodology

A structured literature search was performed in the PubMed database to identify relevant studies examining glutathione (GSH) supplementation, redox balance, and oxidative stress in the context of human immunodeficiency virus (HIV) infection. The search was conducted up to December 2024 using combinations of the following keywords and Boolean operators: “glutathione” AND (“oxidative stress” OR “mitochondrial dysfunction” OR “*N*-acetylcysteine” OR “GlyNAC”) AND (“HIV” OR “human immunodeficiency virus”) AND (“HIV-TB co-infection”).

The initial search yielded 77 articles. Titles and abstracts were independently screened for relevance to glutathione metabolism, oxidative stress, and therapeutic interventions in HIV. Thirty-two articles were excluded due to duplication, lack of relevance to the study objectives, absence of primary data, or insufficient focus on redox or metabolic pathways. The remaining 45 studies were included in the final review.

Inclusion criteria comprised peer-reviewed original research articles, clinical trials, and mechanistic or translational studies involving human participants, animal models, or in vitro systems that evaluated glutathione pathways, redox balance, mitochondrial function, or supplementation strategies in HIV. Studies were required to report measurable biochemical, metabolic, or clinical outcomes related to oxidative stress or antioxidant capacity.

Exclusion criteria included non-English publications, case reports, conference abstracts, editorials, narrative opinions, review articles, and studies lacking quantitative redox, metabolic, or mitochondrial outcome measures. Studies focusing exclusively on unrelated infectious, oncologic, or immunologic outcomes without assessment of oxidative pathways were also excluded.

Reference lists of included studies were manually screened to identify additional relevant publications that met the inclusion criteria.

A narrative synthesis approach was employed due to the substantial heterogeneity among included studies with respect to study design, participant populations, intervention strategies, outcome measures, and analytical methods. This variability precluded formal quantitative meta-analysis. A narrative framework was therefore selected to integrate mechanistic, translational, and clinical evidence, allowing for contextual interpretation of findings and identification of emerging themes across diverse experimental models.

## Data Availability

Data are contained within the article.
